# Antibiofilm activity of a lytic *Salmonella* phage on different *Salmonella enterica* serovars isolated from broiler farms

**DOI:** 10.1007/s10123-022-00294-1

**Published:** 2022-11-05

**Authors:** Reham A. Hosny, Azhar G. Shalaby, Soad A. Nasef, Hend K. Sorour

**Affiliations:** Reference Laboratory for Veterinary Quality Control on Poultry Production, Animal Health Research Institute, Agricultural Research Center, Giza, Egypt

**Keywords:** Bacteriophage, *Salmonella enterica*, Biofilms-gene expression, Microtiter plate

## Abstract

Bacteriophages have been mainly used in treating infections caused by planktonic bacterial cells in the veterinary sector. However, their applications as antibiofilm agents have received little attention. Accordingly, a previously isolated *Salmonella* infecting *Siphoviridae* phage was investigated for host range against 15 *Salmonella enterica *isolates (*S*. Cape, *S*. Gallinarum, 4 *S*. Enteritidis, 3 *S*. Montevideo, *S*. Uno, *S*. Oritamerin, *S*. Belgdam, *S*. Agona, *S*. Daula, and *S*. Aba) recovered from the litters of commercial broiler farms. All *S. enterica *isolates were examined for their biofilm activity using a microtiter plate assay and for *adrA*, *csgD*, and *gcpA* genes using conventional PCR. The phage efficacy against established biofilms produced by the selected seven *S*. *enterica* isolates (*S*. Gallinarum, *S*. Enteritidis, *S*. Montevideo, *S*. Uno, *S*. Oritamerin, *S*. Belgdam, and *S*. Agona) was assessed using microtiter plate assay and reverse transcriptase real-time PCR over different incubation times of 5 and 24 h. All *S. enterica* isolates were strong biofilm formers. Moreover, the phage effectively reduced the biofilm activity of the established *S. enterica* biofilms in the microtiter plate assay using the independent sample *t*-test (*P* < 0.050). Furthermore, the relative expression levels of *csgD*, *gcpA*, and *adrA* genes in the biofilm cells of *S. enterica* isolate after phage treatment were significantly up-regulated to variable degrees using the independent sample *t*-test (*P* < 0.050). In conclusion, the present study revealed the potential use of *Salmonella* phage in reducing established biofilms produced by *S. enterica* serovars isolated from broiler farms.

**Supplementary Information:**

The online version contains supplementary material available at 10.1007/s10123-022-00294-1.

## Introduction

Salmonellosis is one of the most common diseases affecting poultry that has a significant economic impact on the poultry industry and poses a major threat to public health worldwide (Cosby et al. 2015). The genus *Salmonella* consists of more than 2500 serotypes. The diversity of *Salmonella* serotypes reported from poultry sources is based on geographical distribution and time change. However, several serotypes, such as *S*. Enteritidis, *S*. Typhimurium, *S*. Infantis, *S*. Newport, and *S*. Derby, have been reported at a high incidence (Khan et al. 2018; Merino et al. 2019).

Bacterial biofilms are complex microbial communities established on various biotic and abiotic surfaces that are generally enclosed in an extracellular matrix composed of different biopolymers (Hosseinidoust et al., 2013). Biofilm formation can be considered one of the cellular survival mechanisms that make cells more resistant to adverse environmental conditions and provide resistance against different antibiotic intervention regimens (Kirketerp-Møller et al. 2008; Hosseinidoust et al. 2013). Approximately 50% of the *Salmonella* strains isolated from poultry farms produced biofilms in the processing areas of poultry farms and contact surfaces (Wang et al. 2013; Merino et al. 2019). The adhesion of *S*. *enterica* on surfaces is mainly controlled by the presence of biofilm-specific genes such as *csgD*, *adrA*, and *gcpA* related to the synthesis of cellulose and curli fimbriae (García et al. 2004; Bhowmick et al. 2011). The co-expression of fimbriae and cellulose contributes to forming a tightly packed cell matrix enclosed in a hydrophobic network, which is crucial for biofilm formation and tolerance to disinfectants on different surfaces (Jain and Chen 2007). As bacterial biofilms show high antibiotic resistance, eradicating established biofilms is based on using compounds that can penetrate or disrupt them mechanically (Paluch et al. 2020). The most common strategy used for combating microbial adhesion and biofilm formation in poultry houses is based on the chemical attack through cleaning and disinfection procedures. However, these procedures are not fully effective in biofilm eradication (Garcia et al. 2017). The phage-based approach has been studied as an alternative biological tool for biofilm prevention and control (Carson et al. 2010; Kelly et al. 2012). Certain phages can dissolve the extracellular matrix of biofilms by either producing exopolysaccharide-degrading enzymes (EPS-degrading enzymes) such as depolymerases, lysins, DNases, quorum-quenching enzymes, and lipases or inducing the production of EPS-degrading enzymes by bacterial hosts under stress triggered by phage infection ( Maciejewska et al. 2018; Chegini et al. 2020).

Previous investigations have reported the effectiveness of bacteriophages in the inhibition and eradication of the established biofilms produced by clinical *Staphylococcus aureus*, *Pseudomonas aeruginosa*, *Escherichia coli*, *Proteus mirabilis*, and *Klebsiella pneumoniae* (Bedi et al. 2009; Carson et al. 2010; Ahiwale et al. 2011; Kelly et al. 2012)*.*

However, their veterinary applications as antibiofilm agents still have minimal studies. Earlier investigations were mainly concerned with their applications in treating and preventing different infections caused by planktonic cells (Fiorentin et al. 2005; Lau et al. 2010; Nabil et al. 2018).

The results of the only available study by Garcia et al. (2017) have displayed the effectiveness of bacteriophage in the inhibition of biofilm formation in biofilm-forming *S. enterica* serovars (*S*. Enteritidis, *S*. Heidelberg, *S*. Kentucky, *S*. Senftenberg, and *S*. Typhimurium) on surfaces present in chicken slaughterhouses (Garcia et al. 2017). The research on the phage effect on eradicating established *S. enterica* biofilms, including the underlying molecular expression patterns, was given little attention. Therefore, this study aims to provide an evaluation report on the antibiofilm activity of a previously isolated *Salmonella* phage against different strong biofilm former *S. enterica* serovars (*S*. Gallinarum, *S*. Enteritidis, *S*. Montevideo, *S*. Uno, *S*. Oritamerin, *S*. Belgdam, and *S*. Agona) isolated from litters of commercial broiler farms using microtiter plate assay and differential expression of the biofilm-associated genes.

## Materials and methods

### Isolation and identification of S. enterica serovars

Between November 2019 and February 2020, 210 pooled litter samples were collected from 42 commercial broiler farms (five samples/farm) in the Giza governorate. The samples were examined for the isolation of *S. enterica* serovars in the Reference Laboratory for Veterinary Quality Control on Poultry Production, Dokki, Egypt (RLQP, Egypt). The isolation was performed according to ISO6579-1 (2017) by inoculating 10 g of samples into 90 ml of iso buffer peptone water (Oxoid Limited, Thermo Fisher Scientific Inc., UK), and incubating at 37°C for 24 h. After that, 0.1 ml of the enriched sample was inoculated into 9.9 ml of modified semisolid Rappaport-Vassiliadis medium (RV) (LabM, Bury, Lancashire, UK), and incubated at 41.5°C for 24 h. Furthermore, 1 ml of the enriched sample was transferred to Muller-Kauffmann tetrathionate-novobiocin broth (MKTT) (LabM, Bury, Lancashire, UK), and incubated at 37°C for 24 h. A loopful of two tubes was then streaked onto xylose lysine deoxycholate agar (XLD) (LabM, Bury, Lancashire, UK), and brilliant green agar (BG) (Oxoid Limited, Thermo Fisher Scientific Inc., UK) and incubated at 37°C for 24 h. The plates were examined for the presence of red colonies with black centers on XLD agar and red colonies surrounded by a red halo on BG agar. The suspected *Salmonella* colonies were identified according to (ISO6579-1 2017) using different biochemical tests such as triple sugar iron agar, urea, indole, and lysine iron (Oxoid Limited, Thermo Fisher Scientific Inc., UK). Serotyping of *Salmonella* isolates was conducted according to ISO6579-3 (2014) through the identification of somatic (O) and flagellar (H) antigens using SIFIN antisera (Berlin, Germany) located at the RLQP, Egypt.

### Bacteriophage propagation

The phage used in this study was a lytic Siphoviridae *Salmonella* phage previously isolated from poultry litter at a commercial broiler farm in the Giza governorate in 2019 (Sorour et al. 2020).

The phage host propagating strain used in this study was *S*. Kentucky, which was previously isolated from a commercial broiler farm in Giza governorate with severe gross lesions such as enteritis, trachitis, pneumonia, airsacculitis, perihepatitis, pericarditis, nephritis, and typhlitis (Sorour et al. 2020). The phage lysate was amplified, as described by Nabil et al. (2018) and Sorour et al. (2020). Briefly, 4.5 ml of phage lysate (10^8^ PFUml^−1^) was mixed with 0.5 ml mid-exponential *S.* Kentucky culture (OD_600_ = 0.45) and 0.5 ml buffer peptone broth (Oxoid Limited, Thermo Fisher Scientific Inc., UK) in a falcon tube (Techno Plastic Products, Transadingen, Switzerland) and incubated at 37°C for 24 h. The mixture was centrifuged at 11,200 ×*g* for 10 min, and the supernatant was filtered through a 0.45 μm pore syringe filter (Corning, NY, Germany). After that, the phage lysate (10^8^ PFUml^−1^) was tested using tryptic soy agar plates (Oxoid Limited, Thermo Fisher Scientific Inc.) by the spot test and plaque assay as described by Sorour et al. (2020). Phage lysate was then purified three times, as described by Hosny et al. (2021), using the plaque assay method with modification in the broth medium. A single clear plaque was picked into 300 μl of buffer peptone broth, followed by centrifugation and filtration as previously described in the amplification method. The purified phage lysate was stored at 4°C.

### Bacteriophage host range using spot test assay

According to Rahaman et al. (2014), the host range of *Salmonella* phage was tested in vitro against 15 recovered *S. enterica* isolates. Ten microliters of the phage lysate (10^8^ PFUml^−1^) were spotted on tryptic soy agar plates (Oxoid Limited, Thermo Fisher Scientific Inc., UK) overlaid with 15 *S. enterica* isolates. After that, the plates were incubated at 37°C for 24 h and examined for the presence of lytic zones of bacterial growth.

### Bacteriophage efficiency of plating (EOP)

The efficiency of the plating test was determined against 15 recovered *S. enterica* isolates using the plaque assay as described by Vongkamjan et al. (2017) to confirm host-range profiles of *Salmonella* phages obtained from the spot test assay. One hundred microliters of ten folded dilutions of phage lysate (10^1^ to 10^10^) were mixed with 100 µl of the host *S*. Kentucky and the tested isolates. After that, 3 ml of molten soft agar (13 g/l of nutrient broth and 28 g/l of nutrient agar) (HiMedia, Pvt. Ltd., India) were added to the mixture, and overlaid onto tryptic soya agar plates. The titers were determined after 24 h of incubation. Single plaques were counted and expressed as plaque-forming units (PFUml^−1^). EOP was calculated according to the equation: (EOP = average phage titer on the tested isolate/average phage titer on the host isolate). The obtained EOP values were classified as described by Manohar et al. (2019) as high (EOP ≥ 0.5), medium (0.5 < EOP ≥ 0.1), low (0.1 < EOP ˃ 0.001), and inefficient (EOP ≤ 0.001).

### Screening for adrA, gcpA, and csgD biofilm-associated genes in S. enterica isolates using a conventional polymerase chain reaction

The DNAs of 15 *S. enterica* isolates were extracted using a QIAGEN kit (Qiagen, Germany, GmbH). Table S1 summarizes the primer sequences and amplicon sizes used to detect *adrA*, *gcpA*, and *csgD* genes. The specificity of primers was tested using a negative control of *C. perfringens* ATCC 12917 and positive control of a locally isolated *S.* Enteritidis strain containing *adrA*, *gcpA*, and *csgD* genes obtained from the ISO 17025 accredited biotechnology unit, RLQP, Egypt (Nabil and Yonis 2019). Each PCR reaction was performed in a 50 µl total reaction volume containing 25 µl of Emerald Amp® Max PCR master mix (Takara Bio Inc, Japan), 1 µl (20 pmol) of each primer, 11 µl of DNAse and RNAse free water, and 6 µl of genomic DNA extract. The amplification was conducted according to Bhowmick et al. (2011) in an Applied Biosystems 2720 Thermal Cycler (Applied Biosystems). The PCR conditions were 94°C for 5 min for initial denaturation, followed by 30 cycles of denaturation at 94°C for 1 min, annealing at 50°C for *adrA* and *csgD* genes, and 57°C for *gcpA* for 1 min, extension at 72°C for 1 min, and final extension at 72°C for 5 min. The amplified fragments were analyzed in a 1.2% agarose gel stained with 0.2 μg/ml ethidium bromide.

### Detection of the biofilm-forming ability of the S. enterica isolates

A total of 15 *S. enterica* isolates (*S*. cape, *S*. Gallinarum, 4 *S*. Enteritidis, 3 *S*. Montevideo, *S*. Uno, *S*. Oritamerin, *S*. Belgdam, *S*. Agona, *S*. Daula, and *S*. Aba) were examined for their ability for biofilm formation using the microtiter plate assay method. Biofilm formation was quantified using a 96-well flat-bottomed polystyrene microtiter plate (Greiner Bio-One, Germany) as described by Stepanović et al. (2004). Briefly, exponential growth-phase bacteria were prepared by inoculating 10^7^ CFUml^−1^ of each *S. enterica* isolate in Luria-Bertani (LB) broth (Sigma-Aldrich, St. Louis, USA) and incubated at 37°C for 4 h (Spoering and Lewis 2001; Zadeh et al. 2022). After that, 200 μl of bacterial suspensions were added to each well, and the plate was incubated at 37°C for 24 h. Negative control wells contained 200 μl of LB broth alone without bacterial culture. The wells were then poured off and washed three times with sterile phosphate-buffered saline (PBS) (Sigma-Aldrich, St. Louis, USA). The attached bacteria were fixed with 200 μl of 99% methanol (Merck, Darmstadt, Germany) for 15 min, and the wells were then emptied and air-dried. The wells were stained with 200 μl of 0.1% crystal violet solution (Sigma-Aldrich, St. Louis, USA) for 5 min. The stain was rinsed off, and the wells were washed under running tap water. The wells were then left for 15 min for air drying and resolubilized with 200 μl of 33% (v/v) glacial acetic acid. This assay was conducted three times for all *S*. *enterica* isolates and negative control. Each well’s optical density (OD) was measured at 620 nm with an ELISA reader serial no: 610000079 (Tecan Sunrise, Jencons, UK). *S. enterica* isolates were classified into four degrees of biofilm formation based on the optical absorbance of negative control (OD_c_): non-biofilm producer (OD ≤ OD_c_), weak biofilm producer (OD_c_ < OD ≤ 2×OD_c_), moderate biofilm producer (2×OD_c_ < OD ≤ 4×OD_c_), and strong biofilm producer (4×OD_c_ < OD). All measurements were expressed as the mean ± standard deviations.

### Detection of antibiofilm activity of Salmonella phage against established S. enterica biofilms

The evaluation of the phage efficiency in the biofilm control was assessed against established biofilms produced by seven strong biofilm producers of *S. enterica* isolates (*S*. Gallinarum, *S*. Enteritidis, *S*. Montevideo, *S*. Uno, *S*. Oritamerin, *S*. Belgdam, and *S*. Agona) that had OD values of 0.61, 0.78, 0.67, 0.62, 0.61, 0.62, and 0.59, respectively, as described by Chibeu et al. (2012). The filtered phage lysate was diluted in LB broth to obtain five concentrations (10^1^, 10^3^, 10^5^, 10^7^, and 10^9^ PFUml^−1^). Briefly, 200 μl of each diluted phage lysate was added to the established biofilms produced on the microtiter plates by seven *S. enterica* isolates (ten microtiter plates; five plates used for microtiter assay and other five used for gene expression). Negative control wells contained established bacterial biofilms inoculated with 200 μl of LB broth without the addition of phage. After that, the plates were incubated at 37°C for 5 and 24 h to assess the time-dose effect of phage application on the established biofilms. The contents of the wells were transferred into tryptic soy agar plates for phage titration after treatments, and the wells were then washed three times with sterile phosphate-buffered saline (PBS) (Sigma-Aldrich, St. Louis, USA). The remaining biofilm cells were stained as previously described to determine the final biofilm density. The optical density of each well was measured as previously described at 620 nm with an ELISA reader serial no: 610000079 (Tecan Sunrise, Jencons, UK). This assay was conducted three times against all tested *S*. *enterica* isolates and negative controls. All measurements were expressed as the mean ± standard deviations.

### RNA extraction from the established S. enterica biofilms after phage treatment

The phage-treated *S. enterica* biofilms and negative control samples (established bacterial biofilms and LB media without the addition of phage) were scraped three times using a sterile cell scraper (Greiner Bio-One, Germany) from the microtiter plates after 5 and 24 h of treatment and collected in a cold, sterile double-distilled water falcon tube. Samples were then centrifuged for 5 min at 3345 ×*g* at 4°C. Pellets were collected and resuspended in RLT buffer (Qiagen GmbH, Hilden, Germany) and disrupted at high speed (30 Hz) with the Qiagen Tissue Lyser for 2 min. Total RNAs of the collected samples were extracted using the RNeasy Mini Kit (Qiagen) following the manufacturer’s instructions. They were subsequently treated with RNase-Free DNase (Qiagen GmbH, Hilden, Germany) to remove the remaining DNA.

### Quantification of the expression levels of adrA, gcpA, and csgD genes in the established S. enterica biofilms after phage treatment

Reverse transcriptase real-time PCR was used to quantify the level of expression of *csgD*, *adrA*, and *gcpA* genes. The real-time PCR data in this study were represented relative to the 16S rRNA gene as a housekeeping gene (Yang et al. 2014). Primer sequences for amplifying the genes *adrA*, *gcpA*, and *csgD* were outlined in Table S1. Real-time PCR was performed in the Stratagene MX3005P Real-Time System thermal cycler according to Bhowmick et al. (2011) in a total volume of 25 µl, consisting of 12.5 µl of 2x QuantiTect SYBR green master mix (Qiagen, Germany, GmbH), 0.25 µl of RevertAid Reverse Transcriptase (200 U/µl) (Thermo Fisher), 0.5 µl of each primer (20 pmol), 8.25 µl of water, and 3 µl of RNA template. All reactions were performed in triplicates. The thermocycler conditions used for the amplification included an initial reverse transcription at 50°C for 30 min, initial denaturation at 94°C for 15 min followed by 40 cycles of denaturation at 94°C for 15 s, annealing at 60°C for 16s rRNA and 50°C for *adrA* and *csgD* genes, and 57°C for *gcpA* for 30 s, and extension at 72°C for 1 min. A dissociation curve was generated for each gene by denaturation at 94°C for 1 min, annealing at 60°C for 16s rRNA and 50°C for *adrA* and *csgD* genes, and 57°C for *gcpA* for 1 min, and final denaturation at 94°C for 1 min. Data acquisition of the amplification curves and CT values were determined by the Stratagene MX3005P software. Gene expression was calculated as described by Livak and Schmittgen (2001) using the ΔΔCt formula. The expression of the genes was normalized to the 16s rRNA gene as a housekeeping gene by calculating ΔCT. The ΔCT of each sample was compared with that of the negative control group by calculating ΔΔCT. The results of target gene expression were displayed as *n*-fold changes in the transcription level relative to the negative control group.

### Statistical analysis

The data were analyzed using IBM SPSS Statistics (Version 21.0, Armonk, NY, IBM Corp.). All optical density and quantitative real-time PCR data were log_10_-transformed before statistical analyses. An independent sample *t*-test was used to determine the difference in the biofilm eradication and expression levels of biofilm-associated genes between the negative control and the phage-treated groups. The effect of serotyping, phage concentration and their interaction on biofilm eradication was evaluated using a two-way ANOVA with LSD post hoc test. A paired sample *t*-test was used to determine the difference in the biofilm eradication and expression levels of biofilm-associated genes after phage treatment over incubation periods of 5 and 24 h. *P*-values were considered significant at a level ≤ 0.050.

## Results

### Isolation and identification of S. enterica serovars

Among the examined 210 pooled litter samples, the prevalence of salmonellosis was 35.7% (75/210), representing 15 isolates. The biochemical analysis of the suspected *Salmonella* colonies revealed that they were resolved to be *Salmonella* species when they were positive for lysine iron and H_2_S production in triple sugar iron. Meanwhile, they were negative for urea and indole. The identified isolates were *S*. Cape, *S*. Gallinarum, 4 *S*. Enteritidis, 3 *S*. Montevideo, *S*. Uno, *S*. Oritamerin, *S*. Belgdam, *S*. Agona, *S*. Daula, and *S*. Aba (Table 1).

**Table 1:** Host range and efficiency of plating of *Salmonella* phage against *S. enterica* isolates

Bacteriophage host range by spot test		Bacteriophage efficiency of plating				
*S. enterica* isolates tested	Lysis	Host strain	Average bacteriophage titer (PFUml^−1^)	*S. enterica* isolates tested	Average bacteriophage titer (PFUml^−1^)	Efficiency of plating (EOP)
*S*. Cape	+	*S*. Kentucky	143 × 10^8^ ± 0.00	*S*. Cape	160 × 10^8^ ± 0.01	1.1
*S*. Gallinarum	+			*S*. Gallinarum	158 × 10^8^ ± 0.01	1.1
*S*. Enteritidis	+			*S*. Enteritidis	154 × 10^8^ ± 0.02	1.07
*S*. Enteritidis	+			*S*. Enteritidis	152 × 10^8^ ± 0.01	1.06
*S*. Enteritidis	+			*S*. Enteritidis	154 × 10^8^ ± 0.01	1.07
*S*. Enteritidis	+			*S*. Enteritidis	155 × 10^8^ ± 0.02	1.08
*S*. Montevideo	+			*S*. Montevideo	161 × 10^8^ ± 0.01	1.1
*S*. Montevideo	+			*S*. Montevideo	164 × 10^8^ ± 0.01	1.1
*S*. Montevideo	+			*S*. Montevideo	158 × 10^8^ ± 0.02	1.1
*S*. Uno	+			*S*. Uno	160 × 10^8^ ± 0.01	1.1
*S*. Oritamerin	+			*S*. Oritamerin	170 × 10^8^ ± 0.02	1.2
*S*. Belgdam	+			*S*. Belgdam	154 × 10^8^ ± 0.01	1.07
*S*. Agona	+			*S*. Agona	157 × 10^8^ ± 0.01	1.09
*S*. Daula	+			*S*. Daula	153 × 10^8^ ± 0.01	1.03
*S*. Aba	+			*S*. Aba	145 × 10^8^ ± 0.01	1.01

All phage titer values were provided in triplicates and expressed as mean ± standard deviation, *PFU*, Plaque forming unit; EOP was expressed as a ratio of the bacteriophage titer obtained on the tested *S. enterica* strains to that obtained on the host *S*. Kentucky strain

### Bacteriophage host range using spot test assay and efficiency of plating

The *Salmonella* phage showed a broad host range with lytic ability against all *S. enterica* isolates (100%) using a spot test assay. *Salmonella* phage had a high efficiency (˃1) against all *S. enterica* isolates (100%). The EOP of *Salmonella* phage in *S*. Cape, *S*. Gallinarum, *S*. Montevideo, *S*. Uno was 1.1, while in *S*. Enteritidis, it ranged from 1.06 to 1.08. The EOP of *Salmonella* phage in *S*. Oritamerin, *S*. Belgdam, *S*. Agona, *S*. Daula, *S*. Aba was 1.2, 1.07, 1.09, 1.03, and 1.01, respectively (Table 1).

### Screening for adrA, gcpA, and csgD biofilm-associated genes in S. enterica isolates using a conventional polymerase chain reaction

PCR analysis yielded the detection of *adrA* (1113-bp), *csgD* (651-bp), and *gcpA* (1713-bp) genes in all *S. enterica* isolates (Fig. S1a, b, and c).

### Detection of the biofilm-forming ability of the S. enterica isolates using a microtiter plate assay

All *S. enterica* isolates tested in this study produced strong biofilms on a polystyrene microtiter plate. The absorbance ranged from 0.58 to 0.78, and a cutoff value (OD_c_) of 0.12 was used for isolate classification. Isolates were considered strong biofilm formers as the measured OD_620_ was greater than 4-fold the value obtained in the negative control (Table S2).

### Detection of antibiofilm activity of Salmonella phage against established S. enterica biofilms in the microtiter plate assay

**Fig. 1:**
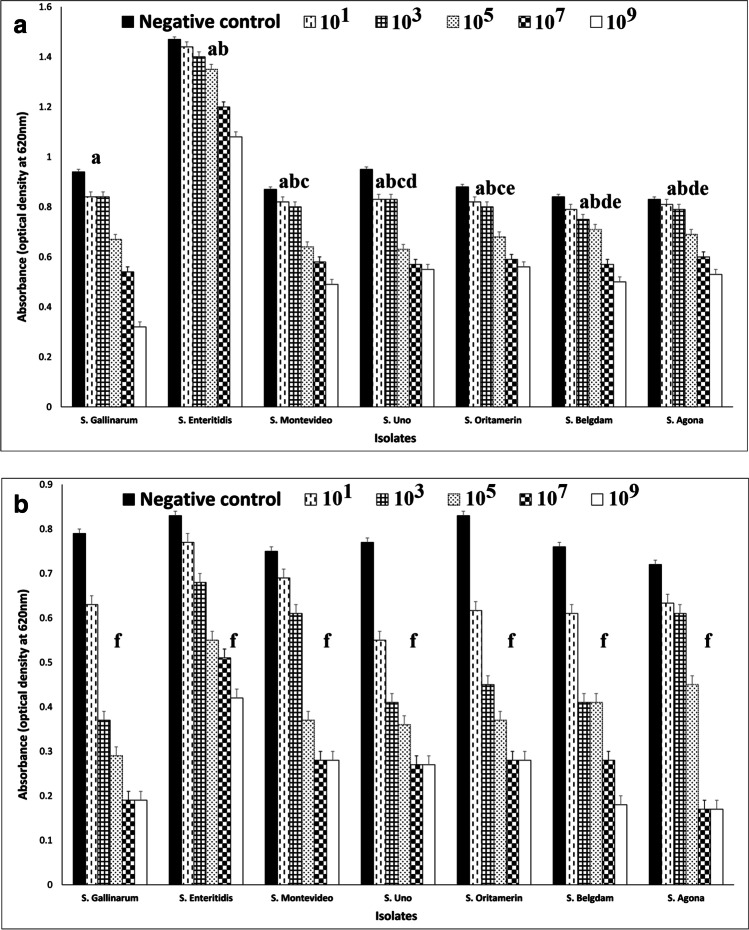
Effect of *Salmonella* phage on the established *S. enterica* biofilms after 5 h (**a**) and 24 h (**b**) of treatment. Phage was effective in the reduction of established biofilms produced by (*S*. Gallinarum, *S*. Enteritidis, *S*. Montevideo, *S*. Uno, *S*. Oritamerin, *S*. Belgdam, and *S*. Agona) compared to untreated negative controls in a time-dose-dependent manner. The data are expressed as mean of three replicated measurements ± standard deviation (represented by error bars). Different letters (a, b, c, d, e, and f) indicate significant differences in the biofilm eradication among isolates based on two-way ANOVA with LSD post hoc test. Isolates that have the same letters are significantly different in the biofilm eradication from each other. P-values were considered significant at a level ≤ 0.050

Overall, the *Salmonella* phage was efficient in a significant reduction in the biofilm mass of established *S. enterica* biofilms over incubation periods of 5 and 24 h in the microtiter plate assay when compared to the negative control group using an independent sample *t*-test (*P* = 0.002 and 0.000, respectively) (Fig. 1a b and Tables S3 and S4). Furthermore, the titers of *Salmonella* phage had increased after 5 and 24 h of treatments at all phage dilutions (10^1^, 10^3^, 10^5^, 10^7^, and 10^9^). This indicates that phage infection and replication were successful (Table S5). A two-way ANOVA was employed to examine the effect of serotyping and phage doses over 5 and 24 h of incubation on biofilm eradication. There was a statistically significant interaction between the effects of serotyping and phage doses on biofilm reduction (*p* = 0.000). Simple main effect analysis using the LSD post hoc test revealed that there were no differences in the biofilm reduction between *S*. Montevideo and *S*. Belgdom (*P* = 0.137), *S*. Montevideo and *S*. Agona (*P* = 0.067), *S*. Oritamerin and *S*. Uno (*P* = 0.616) compared to the remaining *S. enterica* isolates that showed significant differences over an incubation period of 5 h (*P* < 0.050). Furthermore, there were significant differences in the biofilm reduction in all *S. enterica* isolates over an incubation period of 24 h (*P* < 0.050). There were significant differences in the biofilm reduction at all phage concentrations over incubation periods of 5 and 24 h (*P* < 0.050).

The highest mean difference for the biofilm reduction was observed at phage concentration (10^9^ PFUml^−1^) relative to negative control over incubation periods of 5 and 24 h as 0.242 and 0.503, respectively, compared to other phage concentrations (10^1^, 10^3^, 10^5^, and 10^7^ PFUml^−1^). These findings suggest that *Salmonella* phage effectively reduced the established biofilms produced by seven *S*. *enterica* isolates in a dose-dependent manner over incubation periods of 5 and 24 h. A paired sample *t*-test revealed a significant difference in the biofilm reduction after phage treatment over incubation periods of 5 and 24 h (*P* = 0.000). The biofilm reduction after phage treatment over an incubation period of 24 h was 0.245, higher than the incubation period of 5 h.

### Quantification of the expression level of adrA, gcpA, and csgD genes in the established S. enterica biofilms after phage treatment using reverse transcriptase real-time PCR

**Fig. 2:**
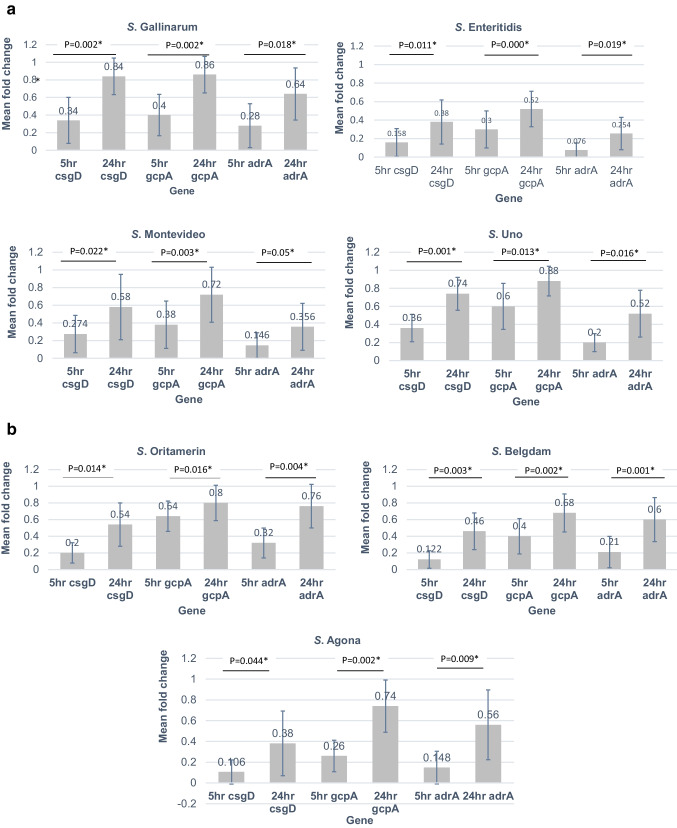
**a**,** b** Relative gene expression of *csgD*, *gcpA* and *adrA* genes in the established *S. enterica* biofilms after 5 and 24 h of phage treatment. A paired sample *t*-test revealed a significant difference in the expression levels of *csgD*, *gcpA* and *adrA* genes after 5 and 24 h of phage treatment (*P* ≤ 0.050). The data are expressed as mean of three replicated measurements for each gene at all phage concentrations ± standard deviation (represented by error bars)

The overall analysis of the biofilm-associated genes (*csgD*, *gcpA*, and *adrA*) in the established *S. enterica* biofilms revealed significant increments at variable degrees in a time-dose-dependent manner after phage treatment over incubation periods of 5 and 24 h compared to the negative control group using an independent sample *t*-test (*P* < 0.050). A paired sample *t*-test revealed a significant difference in the expression levels of biofilm-associated genes (*csgD*, *gcpA*, and *adrA*) after phage treatment over incubation periods of 5 and 24 h (*P* ≤ 0.050) (Fig. 2a, b).

**Fig. 3:**
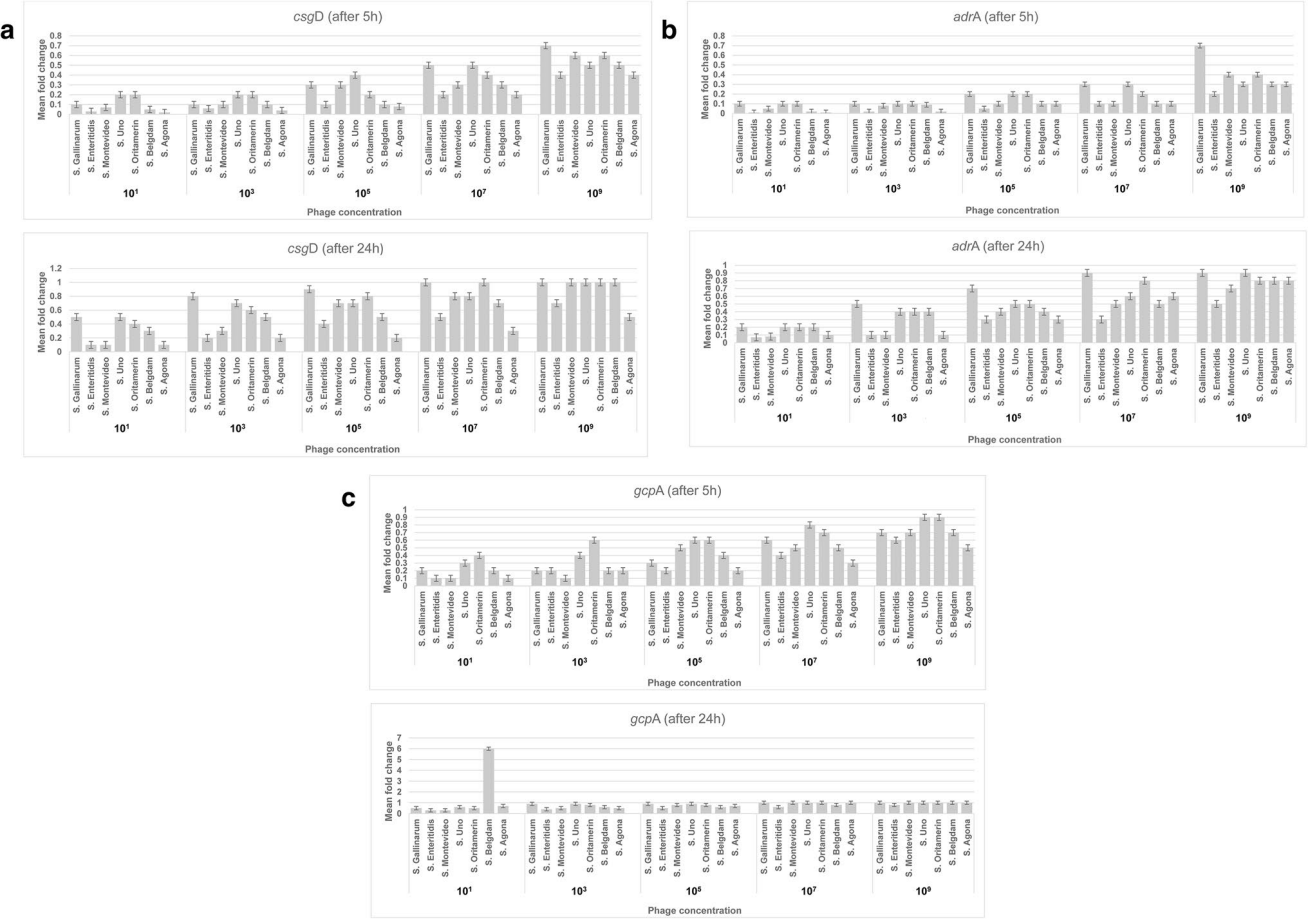
Mean fold change of the expression level of (**a**) *csgD*, (**b**) *adrA*, (**c**) *gcpA* genes in the biofilm forming *S. enterica* isolates treated with different concentrations of phage for 5 and 24 h. Phage was upregulated the expression levels of *csgD*, *adrA*, and *gcpA* genes in the established *S. enterica* biofilms at variable degrees in a time-dose-dependent manner. Fold change was calculated as mean of three replicated measurements ± standard deviation (represented by error bars)

The expression of *csgD* and *adrA* genes in established biofilms produced by six *S*. *enterica* isolates (*S*. Gallinarum, *S*. Enteritidis, *S*. Montevideo, *S*. Oritamerin, *S*. Belgdom, *S*. Agona) following exposure to phage treatment for 5 h revealed elevated levels of expression at concentrations of 10^1^, 10^3^, 10^5^, 10^7^, and 10^9^ PFUml^−1^. Meanwhile, their expression in the established biofilm produced by *S*. Uno increased at concentrations of 10^1^, 10^3^, 10^5^, and 10^7^ PFUml^−1^ with no further increment at 10^9^ PFUml^−1^. Prolonged treatment for 24 h revealed an increase in the expression levels of *csgD* and *adrA* genes in *S*. Enteritidis, *S*. Montevideo, *S*. Belgdom, *S*. Uno, and *S*. Agona at concentrations of 10^1^, 10^3^, 10^5^, 10^7^, and 10^9^ PFUml^−1^ compared to the remaining serotypes, *S*. Gallinarum*,* and *S*. Oritamerin that showed an increase in the expression at concentrations of 10^1^, 10^3^, 10^5^, and 10^7^ PFUml^−1^ with no further increment at 10^9^ PFUml^−1^ (Fig. 3a, b).

The relative expression level of the *gcpA* gene in all established biofilms produced by seven *S*. *enterica* isolates after exposure to phage treatment for 5 h revealed an elevated expression level at concentrations of 10^1^, 10^3^, 10^5^, 10^7^, and 10^9^ PFUml^−1^. Prolonged treatment for 24 h revealed an increase in the expression of the *gcpA* gene in the established biofilms produced by *S*. Enteritidis and S. Belgdom at concentrations of 10^1^, 10^3^, 10^5^, 10^7^, and 10^9^ PFUml^−1^ compared to the remaining serotypes, *S*. Gallinarum, *S*. Montevideo, *S*. Uno, *S*. Agona, and *S*. Oritamerin that showed an increase in the expression at concentrations of 10^1^, 10^3^, 10^5^, and 10^7^ PFUml^−1^, with no further increment at 10^9^ PFUml^−1^ (Fig. 3c).

## Discussion

Bacteriophages are viruses infecting bacteria identified as antibiotic alternatives (Dalmasso et al. 2016). Earlier clinical studies have displayed the potential use of bacteriophages in biofilm control and eradication; however, their applications as antibiofilm agents in the veterinary field have not been reported (Bedi et al. 2009; Carson et al. 2010; Ahiwale et al. 2011; Kelly et al. 2012).

The* Salmonella* phage used in this study has previously been isolated, characterized, and assessed for its potential role in the prevention and treatment of *S*. Kentucky infection in broilers (Sorour et al., 2020). However, this *Salmonella* phage has never been evaluated for the treatment of established biofilms produced by different *S. enterica* serovars, and thus, this is worthy of our investigation.

In this study, ten different *Salmonella* serovars were identified (*S*. Cape, *S*. Gallinarum, *S*. Enteritidis, *S*. Montevideo, *S*. Uno, *S*. Oritamerin, *S*. Belgdam, *S*. Agona, *S*. Daula, and *S*. Aba). The most prevalent *Salmonella* serovars were *S*. Enteritidis (26.7%) and *S*. Montevideo (20%). Similarly, previous studies conducted by Cheong et al. (2007) and Kim et al. (2012) have displayed a high incidence of several *S*. *enterica* serotypes in broiler chickens, such as *S*. Enteritidis (21.9% and 57.4% ), *S*. Typhimurium (23.4% and 6.4%), *S*. Montevideo (9.4% and 31.9%), respectively (Cheong et al. 2007; Kim et al. 2012). Furthermore, earlier research has reported that some *Salmonella* serotypes, such as *S*. Enteritidis, *S*. Typhimurium, *S*. Infantis, *S*. Newport, and *S*. Derby are the most recovered ones associated with poultry-associated infections (Álvarez-Fernández et al. 2012; Khan et al. 2018). Our study provided evidence of the prevalence of *Salmonella* in the litter samples (35%). This finding is consistent with a previous study by Shang et al. (2018). They reported that environmental samples such as poultry litter, feed, air, and fans are important reservoirs for *Salmonella* infections in poultry farms.

The lytic profile of the *Salmonella* phage was determined against 15 *S.* *enterica* isolates using spot test and the efficiency of plating assay. The phage lysed all *S. enterica* isolates (*S*. Cape, *S*. Gallinarum, 4 *S*. Enteritidis, 3 *S*. Montevideo, *S*. Uno, *S*. Oritamerin, *S*. Belgdam, *S*. Agona, *S*. Daula, and *S*. Aba) with high efficiency of plating (˃1). The broad host range characteristic of *Salmonella* phage observed in this study suggests its suitability for biocontrol applications. According to Bielke et al. (2007), not all bacteriophages are host-specific and genera-restricted. This may be useful for selecting bacteriophages with broad host ranges for the pathogen of interest. Pelyuntha et al. (2021) reported that *Salmonella* phages WP109 and 110 showed high EOP on *S*. Kentucky, *S*. Saintpaul, *S*. Enteritidis, and *S*. Albany*.* Gomez-Garcia et al. (2021) showed that *Salmonella* phage S1 showed high EOP on *S.* Pullorum, *S*. Gallinarum, and *S*. Enteritidis. Esmael et al. (2021) demonstrated that *Salmonella* phage cocktails SPHG1 and SPHG3 showed high EOP against all *S*. Typhimurium tested. Mahmoud et al. (2018) reported the ability of *Siphoviridae* bacteriophages (Salmacey1 and Salmacey2) to lyse four different *Salmonella* serovars; *S*. Typhimurium, *S*. Enteritidis, *S*. Kentucky, and *S*. Typhi using a spot test assay. Furthermore, Bielke et al. (2007) demonstrated the ability of a wide host range phage (WHR 8) to lyse 6 different *Salmonella* serovars; *S.* Montevideo,* S.* Heidelberg,* S.* Ohio, *S.* Typhimurium*, S.* Agona*,* and *S.* Minnesota using a spot test assay.

All *S. enterica *isolates were examined for their biofilm-forming activity using a microtiter plate assay and conventional PCR. The microtiter plates used in the biofilm assay mimic plastic materials commonly used in poultry farms (Borges et al. 2018). Our results showed that all *S. enterica* isolates could produce strong biofilm on a polystyrene microtiter plate. These results agree with earlier studies that reported the adherence of S. *enterica* serotypes to hydrophobic surfaces such as polystyrene (Tondo et al. 2010; Borges et al. 2018). Agarwal et al. (2011) demonstrated that intrinsic characteristics in *Salmonella* serovars, such as fimbriae, flagella, membrane proteins, and other cellular appendages, play a role in their biofilm activity. Borges et al. (2018) displayed the ability of *S.* Agona and *S.* Montevideo serotypes to be good biofilm producers. Molecular characterization revealed positive amplification for (*adrA*,* csgD*, and *gcpA*) genes in all examined *S. enterica* isolates. The selection of these genes in this study was based on results of earlier studies that reported the presence of biofilm-specific genes, such as *csgD*, *adrA*, and *gcpA* that play a role in the adherence of *S. enterica* on surfaces through involvement in the production of cellulose and the expression of curli fimbriae (García et al. 2004; Bhowmick et al. 2011). Bhowmick et al. (2011) reported similar observations regarding detecting these genes in different *Salmonella* serovars, such as *S*. Weltevreden, *S*. Enteritidis, and *S*. Typhimurium.

The *Salmonella* phage action was assessed in different concentrations against established biofilms produced by seven strong biofilm formers *S. enterica* isolates (*S*. Gallinarum, *S*. Enteritidis, *S*. Montevideo, *S*. Uno, *S*. Oritamerin, *S*. Belgdam, and *S*. Agona) using a microtiter plate assay over incubation times of 5 and 24 h. A time-dose-dependent reduction in the established biofilms produced by seven *S. enterica* isolates was observed over incubation for 5 and 24 h of treatment. It could mean that phage’s high titer (10^9^ PFUml^−1^) was the most effective in reducing established biofilms after 5 and 24 h of treatment. Similarly, a study by Cornelissen et al. (2011) has displayed a time-dose-dependent reduction in *Pseudomonas putida* biofilm mass with a maximum reduction of 8 h after the addition of the high φ15 phage doses (10^4^ and 10^6^ PFUml^−1^). A study by Mapes et al. (2016) has displayed a significant reduction of clinically established *Pseudomonas aeruginosa* biofilms in a dose-dependent manner after 6 h of exposure to phage cocktail treatment.

On the other hand, Chibeu et al. (2012) demonstrated that vB_EcoP_ACG-C91, vB_EcoM_ACG-C40, and vB_EcoS_ACG-M12 phages significantly reduced the established biofilms produced by uropathogenic *Escherichia coli* over treatment for 8 h in a dose-independent manner. A study by Cerca et al. (2007) has demonstrated that *Staphylococcus* bacteriophage K (10^8^ PFUml^−1^) significantly reduced established *S*. *epidermidis* biofilms after 24 h of treatment. Until now, there have been no published reports on the antibiofilm activity of *Salmonella* phage against *S. enterica* serotypes recovered from poultry samples. This study observed that phage was not equally eradicating the established biofilms produced by *S. enterica* isolates based on LSD post hoc test results. This might be attributed to the variation in the serotypes. Phage treatment of biofilms for 24 h resulted in a reduction of the established biofilms produced by *S*. Enteritidis and *S.* Belgdam at concentrations of 10^1^, 10^3^, 10^5^, 10^7^, and 10^9^ PFUml^−1^ compared to the remaining serotypes, *S*. Gallinarum, *S*. Montevideo, *S*. Uno, *S*. Agona, and *S*. Oritamerin that showed a reduction of the established biofilms at concentrations of 10^1^, 10^3^, 10^5^, and 10^7^ PFUml^−1^, with no further decrease at 10^9^ PFUml^−1^. These findings may indicate the re-establishment of the biofilms at higher phage concentrations ˃ 10^9^ PFUml^−1^ over an incubation time of 24 h. Previous studies have displayed an increase in the biofilm mass produced by *Pseudomonas aeruginosa* and uropathogenic *E. coli* after 24 h of phage treatment. This may be attributed to the development of phage-resistant variants (Pires et al. 2011; Chibeu et al. 2012). Chibeu et al. (2012) reported that combining phage treatment with chemical antimicrobials could be an effective technique for preventing phage-resistant cells from re-establishing biofilms.

The quantification of specific messenger RNA was evaluated by reverse transcriptase real-time PCR to assess specific changes in the expression patterns of the biofilm-associated genes (*adrA*, *csgD*, and *gcpA*) in the established *S. enterica* biofilms after phage treatment. The molecular findings revealed that the phage treatment for 5 and 24 h up-regulated the expression of *adrA*,* csgD*, and *gcpA* genes in a time-dose-dependent manner in the established biofilms produced by seven *S. enterica* isolates. It could mean that the highest expression level was reported at phage titer (10^9^ PFUml^−1^). This might relate to the bacterial survival levels in biofilms that could be explained by the presence of persistent cells within the biofilm that increased by an increment of phage concentration. This possibility complies with the microtiter plate assay results that revealed the phage’s ability to reduce, not eradicate, the established *S. enterica* biofilms over 5 and 24 h. Biofilms represent a potential reservoir for persistent bacteria, a non-heritable phenotypic variation in the bacterial population that displays high antibiotic tolerance by entering a slow-growth or non-growth state (Harms et al. 2017; Drescher et al. 2019). A study by Drescher et al. (2019) reported that persistent cells could spontaneously arise in a microbial population or be triggered by stressors such as antimicrobials. The formation of persistent cells is mediated by different mechanisms that either act alone or overlapped, such as stress response, toxin-antitoxin, energy production, and phosphate metabolism (Drescher et al. 2019).

Furthermore, previous studies have displayed that the number of persistent cells in biofilm increased in the older biofilms compared to the younger ones (Abedon 2016; Ferriol-Gonzalez and Domingo-Calap 2020). Accordingly, the suggestion of the presence of persistent cells within the biofilm in this study may be attributed to the response mechanism of bacteria to the phage treatment and the old biofilm age. Harper et al. (2014) reported that bacteriophages could infect persistent cells but remain dormant until they revert to normal cells and initiate a productive lytic infection. Therefore, phages can target persistent cells within biofilm through antibiotic treatment (Chegini et al. 2020). Li et al. (2021) reported that combining phage with antibiotic treatment can significantly destroy the bacterial biofilms, release the persistent cells into the nutrient environment, and subsequently enhance the metabolic activity of persistent cells, making them more sensitive to the phage treatment.

## Study limitations

The limitation of this study is that the antibiofilm activity of the phage was assessed against only seven *S. enterica* isolates; this was attributed to the scarce resources in our country. Moreover, the limitations of the molecular method in this study are the lack of assessment of the molecular mechanisms behind the persistent cell formation and other biofilm-associated genes such as *csgA*, *BcsA*, *RpoS*, *CrL*, *OmpR*, and *MlrA* that play a role in the production of cellulose and the expression of curli fimbriae in *S*. *enterica* species (Chen et al. 2021).

## Conclusions

The present study has displayed the potential use of *Salmonella* phage in reducing established biofilms produced by *S. enterica* serovars isolated from broiler farms. Furthermore, this study is the first to identify transcriptional expression patterns of the biofilm-associated genes after phage treatment. Further research studies are needed to assess the efficacy of *Salmonella* phage in preventing biofilm formation for comparison with the results of this study that have assayed its efficacy in eradicating established biofilms.

## Supplementary Information

Below is the link to the electronic supplementary material.

Supplementary file1 (TIF 3021 KB)

Supplementary file2 (TIF 2787 KB)

Supplementary file3 (TIF 3149 KB)

Supplementary file4 (DOCX 16 KB)

Supplementary file5 (DOCX 15 KB)

Supplementary file6 (DOCX 17 KB)

Supplementary file7 (DOCX 17 KB)

Supplementary file8 (DOCX 17 KB)

## Data Availability

The authors confirm that the data supporting the findings of this study are available within the article and its supplementary information.
