# Supplementary figures and images for: Antibiofilm activity of a lytic Salmonella phage on different Salmonella enterica serovars isolated from broiler farms
Source: Int Microbiol. 2022 Nov 5;26(2):205–17. doi: 10.1007/s10123-022-00294-1 (PMC10148789; doi:10.1007/s10123-022-00294-1)

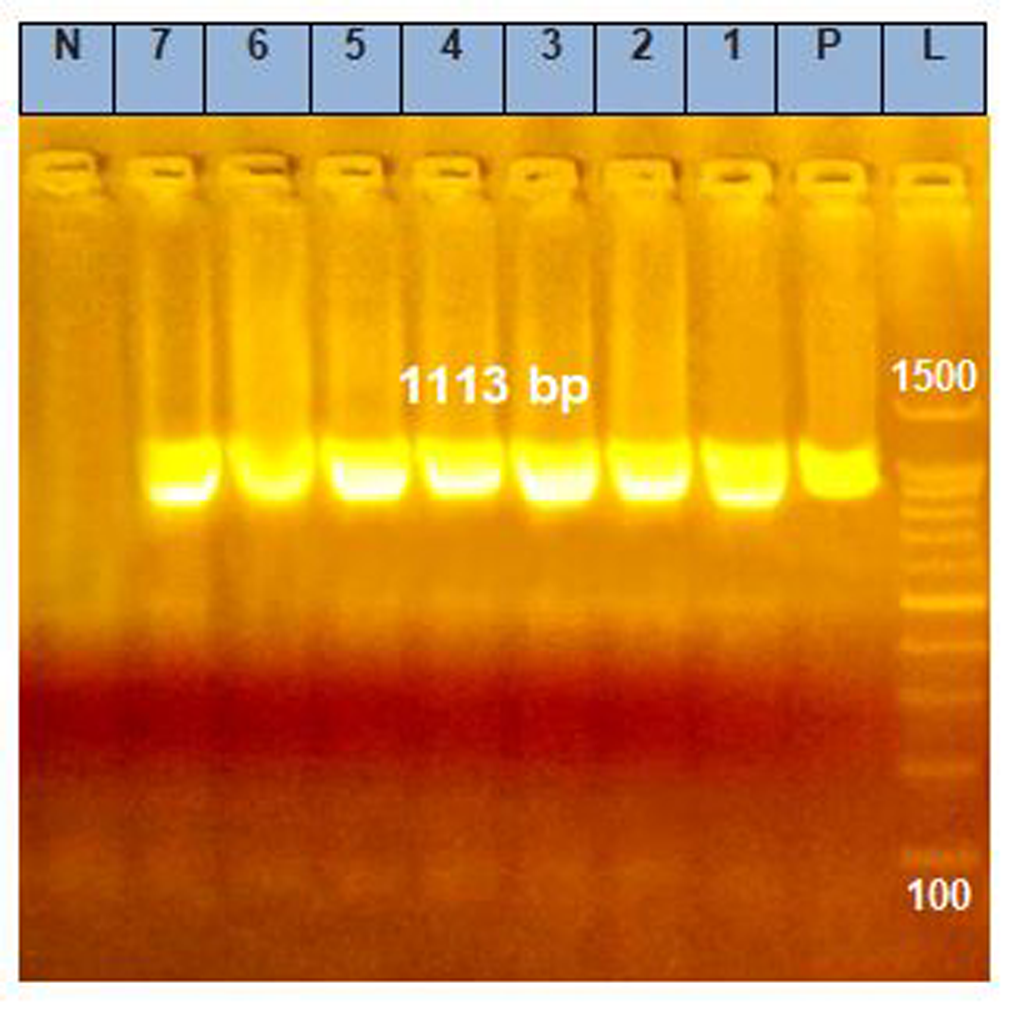

Supplement: Supplementary file 1 — Supplementary file1 (TIF 3021 KB) [file 10123_2022_294_MOESM1_ESM.tif]

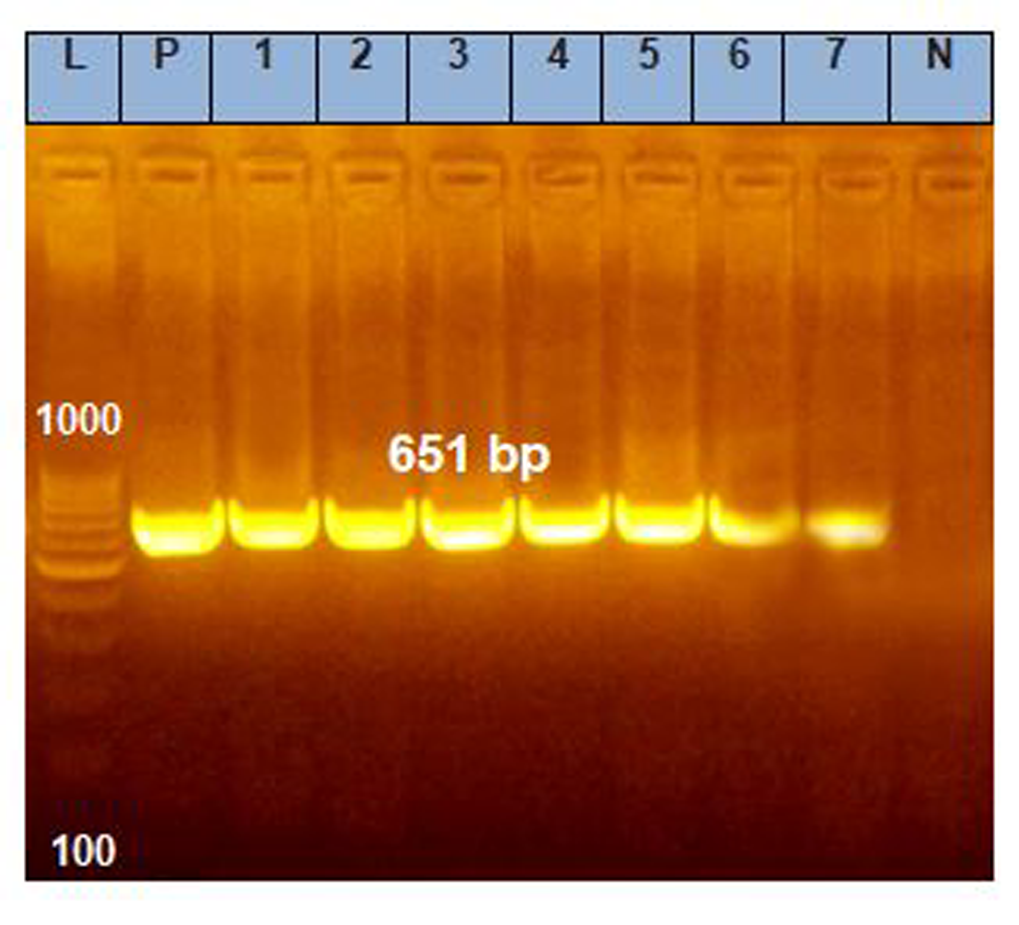

Supplement: Supplementary file 2 — Supplementary file2 (TIF 2787 KB) [file 10123_2022_294_MOESM2_ESM.tif]

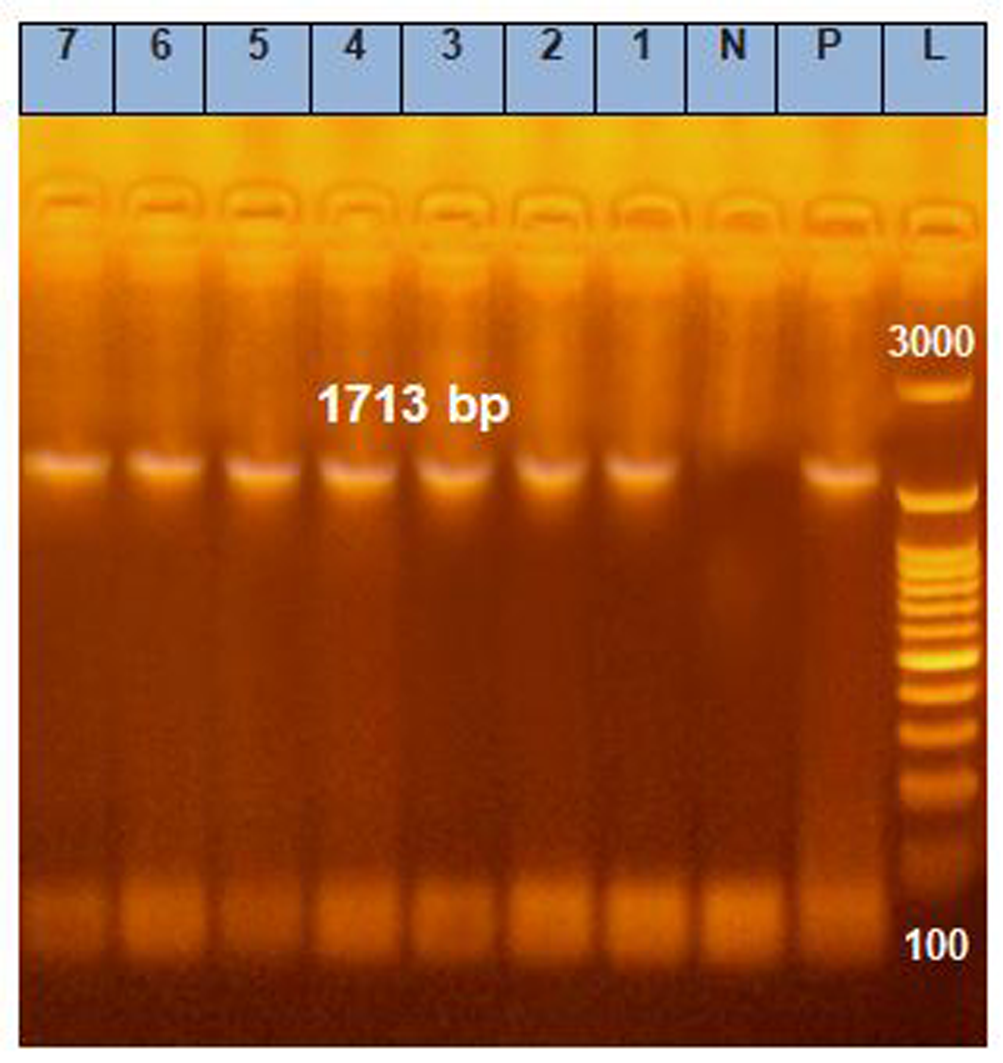

Supplement: Supplementary file 3 — Supplementary file3 (TIF 3149 KB) [file 10123_2022_294_MOESM3_ESM.tif]
