# Supplementary material for: Antibiofilm activity of a lytic Salmonella phage on different Salmonella enterica serovars isolated from broiler farms
Source: Int Microbiol. 2022 Nov 5;26(2):205–17. doi: 10.1007/s10123-022-00294-1 (PMC10148789; doi:10.1007/s10123-022-00294-1)
Supplement: Supplementary file 4 — Supplementary file4 (DOCX 16 KB) [file 10123_2022_294_MOESM4_ESM.docx]

**Table S1. Primers used for the detection of *adr*A, *gcp*A, and *csg*D genes in *S. enterica* isolates using conventional and reverse transcriptase real-time PCR.**

| **Target genes** | **Primers sequences** | **Reference** | **Type of PCR** | **Length of amplified product (bp)** | |
| --- | --- | --- | --- | --- | --- |
|  |  |  |  |  |  |
| ***adr*A** | ATGTTCCCAAAAATAATGAA | Bhowmick et al. 2011 | Conventional PCR | 1113 | |
|  | TCATGCCGCCACTTCGGTGC |  |  |  |  |
| ***gcp*A** | CTATTTCTTTTCCCGCTCCT |  |  | 1713 | |
|  | GTGCCGCACGAAACACTGTT |  |  |  |  |
| ***csg*D** | TTACCGCCTGAGATTATCGT |  |  | 651 | |
|  | ATGTTTAATGAAGTCCATAG |  |  |  |  |
| ***16S rRNA*** | CAGAAGAAGCACCGGCTAACTC | Yang et al. 2014 | Reverse- transcriptase real time PCR | | 87 |
|  | GCGCTTTACGCCCAGTAATT |  |  |  |  |
| ***adr*A** | CGCTGGAAGTCACGCTCTCT | Bhowmick et al. 2011 |  |  | 102 |
|  | GTCGCTTATGTTCCGCTAATTTAAT |  |  |  |  |
| ***gcp*A** | GCGTCATTACAGCGGTTCCT |  |  |  | 80 |
|  | CGGAGATGGTTATCGTATAGCTGAT |  |  |  |  |
| ***csg*D** | CGCCACGCAGAATACCCT |  |  |  | 72 |
|  | TTAACGGCGTGTTTTACGCTAC |  |  |  |  |
