# Supplementary material for: Antibiofilm activity of a lytic Salmonella phage on different Salmonella enterica serovars isolated from broiler farms
Source: Int Microbiol. 2022 Nov 5;26(2):205–17. doi: 10.1007/s10123-022-00294-1 (PMC10148789; doi:10.1007/s10123-022-00294-1)
Supplement: Supplementary file 5 — Supplementary file5 (DOCX 15 KB) [file 10123_2022_294_MOESM5_ESM.docx]

**Table S2. Biofilm-forming ability of *S. enterica* isolates**

| Isolates | **Optical density** | **Biofilm status** |
| --- | --- | --- |
|  | **Means ± standard deviation** |  |
| Negative control | 0.12 **±** 0.02 |  |
| *S*. Cape | 0.63 **±** 0.03 | strong |
| *S*. Gallinarum | 0.61 **±** 0.05 | strong |
| *S*. Enteritidis | 0.78 **±** 0.06 | strong |
| *S*. Montevideo | 0.67 **±** 0.01 | strong |
| *S*. Daula | 0.58 **±** 0.01 | strong |
| *S*. Uno | 0.62 **±** 0.05 | strong |
| *S*. Aba | 0.60 **±** 0.02 | strong |
| *S*. Enteritidis | 0.63 **±** 0.02 | strong |
| *S*. Oritamerin | 0.61 **±** 0.05 | strong |
| *S*. Montevideo | 0.58 **±** 0.02 | strong |
| *S*. Enteritidis | 0.64 **±** 0.01 | strong |
| *S*. Belgdom | 0.62 **±** 0.03 | strong |
| *S*. Agona | 0.59 **±** 0.01 | strong |
| *S*. Enteritidis | 0.64 **±** 0.02 | strong |
| *S*. Montevideo | 0.60 **±** 0.01 | strong |

The optical density measurements are expressed as mean of three replicated measurements ± standard deviation
