# Supplementary material for: Antibiofilm activity of a lytic Salmonella phage on different Salmonella enterica serovars isolated from broiler farms
Source: Int Microbiol. 2022 Nov 5;26(2):205–17. doi: 10.1007/s10123-022-00294-1 (PMC10148789; doi:10.1007/s10123-022-00294-1)
Supplement: Supplementary file 6 — Supplementary file6 (DOCX 17 KB) [file 10123_2022_294_MOESM6_ESM.docx]

**Table S3. Antibiofilm effect of different *Salmonella* phage concentrations after 5 hours of treatment.**

| **Isolates** | **Optical density** | | | | | |
| --- | --- | --- | --- | --- | --- | --- |
|  | **Negative control** | **Phage concentrations** | | | | |
|  |  | **10^1^** ^f^ | **10^3^** ^f^ | **10^5^** ^f^ | **10^7^** ^f^ | **10^9^** ^f^ |
| *S*. Gallinarum ^a^ | 0.94±0.006 | 0.84±0,01 | 0.84±0.001 | 0.67±0.023 | 0.54±0.006 | 0.32±0.021 |
| *S*. Enteritidis ^ab^ | 1.47±0.01 | 1.44±0.01 | 1.40±0.006 | 1.35±0.061 | 1.20±0.017 | 1.08±0.01 |
| *S*. Montevideo ^abc^ | 0.87±0.006 | 0.82±0.02 | 0.80±0.02 | 0.64±0.01 | 0.58±0.021 | 0.49±0.015 |
| *S*. Uno ^abcd^ | 0.95±0.02 | 0.83± 0.02 | 0.83±0.02 | 0.63±0.01 | 0.57±0.01 | 0.55±0.01 |
| *S*. Oritamerin ^abce^ | 0.88±0.01 | 0.82±0.01 | 0.80±0.006 | 0.68±0.01 | 0.59±0.01 | 0.56±0.012 |
| *S*. Belgdam ^abde^ | 0.84±0.026 | 0.79±0.006 | 0.75±0.006 | 0.71±0.01 | 0.57±0.01 | 0.50±0.01 |
| *S*. Agona ^abde^ | 0.83±0.006 | 0.81±0.017 | 0.79±0.069 | 0.69±0.006 | 0.60±0.01 | 0.53±0.006 |
| **Independent sample t test** | P= 0.002 | | | | | |
| **Two-way Anova** | Isolates: F= 2197.149, P= 0.000  Phage concentrations: F= 1970.876, P=0.000  Interaction: F= 62.280, P=0.000  Mean difference in the biofilm eradication based on treatment:  Negative control- 10^1^=0.031  Negative control- 10^3^= 0.039  Negative control- 10^5^= 0.109  Negative control- 10^7^= 0.170  Negative control- 10^9^= 0.242 | | | | | |

All optical density measurements were performed in triplicates and expressed as mean ± standard deviation, Different letters (a,b,c,d,e) indicate significant differences in the biofilm eradication among isolates based on LSD post-hoc test. Letter f indicates significant differences in the biofilm eradication among phage concentrations based on LSD post-hoc test. Isolates and phage concentrations that have the same letters are significantly different in the biofilm eradication from each other. P-values were considered significant at a level ≤ 0.050.

# 
