# Supplementary material for: Antibiofilm activity of a lytic Salmonella phage on different Salmonella enterica serovars isolated from broiler farms
Source: Int Microbiol. 2022 Nov 5;26(2):205–17. doi: 10.1007/s10123-022-00294-1 (PMC10148789; doi:10.1007/s10123-022-00294-1)
Supplement: Supplementary file 7 — Supplementary file7 (DOCX 17 KB) [file 10123_2022_294_MOESM7_ESM.docx]

**Table S4. Antibiofilm effect of different *Salmonella* phage concentrations after 24 hours of treatment.**

| **Isolates** | **Optical density** | | | | | |
| --- | --- | --- | --- | --- | --- | --- |
|  | **Negative control** | **Phage concentrations** | | | | |
|  |  | **10^1^** ^b^ | **10^3^** ^b^ | **10^5^** ^b^ | **10^7^** ^b^ | **10^9^** ^b^ |
| *S*. Gallinarum ^a^ | 0.79±0.01 | 0.63±0.02 | 0.37±0.017 | 0.29±0.02 | 0.19±0.02 | 0.19±0.01 |
| *S*. Enteritidis ^a^ | 0.83±0.006 | 0.77±0.02 | 0.68±0.023 | 0.55±0.006 | 0.51±0.01 | 0.42±0.006 |
| *S*. Montevideo ^a^ | 0.75±0.02 | 0.69±0.006 | 0.61±0.02 | 0.37±0.01 | 0.28±0.01 | 0.28±0.006 |
| *S*. Uno ^a^ | 0.77±0.026 | 0.55± 0.01 | 0.41±0.01 | 0.36±0.01 | 0.27±0.02 | 0.27±0.017 |
| *S*. Oritamerin ^a^ | 0.83±0.01 | 0.62±0.01 | 0.45±0.01 | 0.37±0.01 | 0.28±0.01 | 0.28±0.02 |
| *S*. Belgdam ^a^ | 0.76±0.006 | 0.61±0.006 | 0.41±0.006 | 0.41±0.01 | 0.28±0.02 | 0.18±0.01 |
| *S*. Agona ^a^ | 0.72±0.01 | 0.63±0.01 | 0.61±0.01 | 0.45±0.017 | 0.17±0.01 | 0.17±0.01 |
| **Independent sample t test** | P= 0.000 | | | | | |
| **Two-way Anova** | Isolates: F= 2251.989, P= 0.000  Phage concentrations: F= 18441.747, P=0.000  Interaction: F= 384.482, P=0.000  Mean difference in the biofilm eradication based on treatment:  Negative control- 10^1^=0.085  Negative control- 10^3^=0.197  Negative control- 10^5^=0.297  Negative control- 10^7^=0.465  Negative control- 10^9^=0.503 | | | | | |
| **Paired sample t test** | Mean difference=0.245, t= -20.866, p=0.000 | | | | | |

All optical density measurements were performed in triplicates and expressed as mean ± standard deviation, letter a indicates significant differences in the biofilm eradication among isolates based on LSD post-hoc test. Letter b indicates significant differences in the biofilm eradication among phage concentrations based on LSD post-hoc test. P-values were considered significant at a level ≤ 0.050.
