# Supplementary material for: Antibiofilm activity of a lytic Salmonella phage on different Salmonella enterica serovars isolated from broiler farms
Source: Int Microbiol. 2022 Nov 5;26(2):205–17. doi: 10.1007/s10123-022-00294-1 (PMC10148789; doi:10.1007/s10123-022-00294-1)
Supplement: Supplementary file 8 — Supplementary file8 (DOCX 17 KB) [file 10123_2022_294_MOESM8_ESM.docx]

**Table S5. Phage titration after 5- and 24-hours of treatment**

| ***S. enterica* isolates** | **Post- treatment phage titers (mean log PFUml^-1^ ± standard deviation)** | | | | | | | | | |
| --- | --- | --- | --- | --- | --- | --- | --- | --- | --- | --- |
|  | **after 5 hours of treatment** | | | | | **after 24 hours of treatment** | | | | |
|  | **10^1^** | **10^3^** | **10^5^** | **10^7^** | **10^9^** | **10^1^** | **10^3^** | **10^5^** | **10^7^** | **10^9^** |
| ***S*. Gallinarum** | 2.5±  0.01 | 4.3±  0.01 | 6.3±  0.02 | 8.5± 0.02 | 10.3±  0.01 | 3.3± 0.02 | 5.6± 0.02 | 7.5±  0.01 | 9.5±  0.01 | 11.3±  0.01 |
| ***S*. Enteritidis** | 2.8±0.02 | 4.8±  0.01 | 6.8± 0.015 | 8.8±  0.01 | 10.8±0.006 | 3.9± 0.02 | 5.9±  0.01 | 7.9 ±0.02 | 9.9 ±0.02 | 11.8±0.017 |
| ***S*. Montevideo** | 3.6±0.02 | 5.4±0.02 | 7.1±  0.01 | 9.2±  0.01 | 11.0±0.02 | 4.7±  0.01 | 6.5±  0.01 | 8.4 ±0.017 | 10.2±  0.01 | 12.4±  0.02 |
| ***S*. Uno** | 3.7±  0.01 | 5.7± 0.015 | 7.5 ±0.02 | 9.4±  0.02 | 11.2±  0.01 | 4.8± 0.006 | 6.7 ±0.02 | 8.6±  0.02 | 10.7 ±0.02 | 12.9±0.021 |
| ***S*. Oritamerin** | 3.9±  0.01 | 5.9±  0.01 | 8.0± 0.017 | 9.9 ±  0.01 | 11.9 ±0.017 | 5.0± 0.02 | 7.0±  0.01 | 8.9±  0.01 | 10.9±  0.01 | 13.0±  0.01 |
| ***S*. Belgdam** | 2.7±  0.01 | 4.7±0.02 | 6.8±  0.01 | 8.7±  0.02 | 10.8±0.021 | 3.8±  0.01 | 5.8±  0.01 | 7.8±  0.01 | 9.8± 0.021 | 11.6±  0.01 |
| ***S*. Agona** | 2.9±  0.02 | 5.0 ±  0.01 | 7.0±  0.01 | 9.0±  0.01 | 11.0±  0.01 | 4.0±  0.01 | 6.0±  0.016 | 8.0±  0.01 | 10.0±  0.01 | 11.8±  0.01 |

All phage titers were measured in triplicates and expressed as mean log PFUml^-1^ ± standard deviation
